# Developments in high-resolution mass spectrometric analyses of new psychoactive substances

**DOI:** 10.1007/s00204-022-03224-2

**Published:** 2022-02-09

**Authors:** Joshua Klingberg, Bethany Keen, Adam Cawley, Daniel Pasin, Shanlin Fu

**Affiliations:** 1 Australian Racing Forensic Laboratory, Racing NSW, Sydney, NSW 2000 Australia; 2 Centre for Forensic Science, University of Technology Sydney, Broadway, NSW 2007 Australia; 3 Section of Forensic Chemistry, Department of Forensic Medicine, University of Copenhagen, Copenhagen, Denmark

**Keywords:** New psychoactive substances, Illicit drugs, HRMS, Machine learning, Molecular networking, Metabolomics

## Abstract

The proliferation of new psychoactive substances (NPS) has necessitated the development and improvement of current practices for the detection and identification of known NPS and newly emerging derivatives. High-resolution mass spectrometry (HRMS) is quickly becoming the industry standard for these analyses due to its ability to be operated in data-independent acquisition (DIA) modes, allowing for the collection of large amounts of data and enabling retrospective data interrogation as new information becomes available. The increasing popularity of HRMS has also prompted the exploration of new ways to screen for NPS, including broad-spectrum wastewater analysis to identify usage trends in the community and metabolomic-based approaches to examine the effects of drugs of abuse on endogenous compounds. In this paper, the novel applications of HRMS techniques to the analysis of NPS is reviewed. In particular, the development of innovative data analysis and interpretation approaches is discussed, including the application of machine learning and molecular networking to toxicological analyses.

## Introduction

The United Nations Office on Drugs and Crime (UNODC) defines new psychoactive substances (NPS) as ‘substances of abuse, either in a pure form or a preparation, that are not controlled by the 1961 Single Convention on Narcotic Drugs or the 1971 Convention on Psychoactive Substances, but which may pose a public health threat’ (United Nations Office on Drugs and Crime 2021). In this context, the word ‘new’ does not necessarily mean new inventions, but merely substances that have recently appeared on the drug market (United Nations Office on Drugs and Crime 2021). These substances are often marketed as ‘legal highs’, ‘bath salts’, or ‘research chemicals’ in order to circumvent legislation (United Nations Office on Drugs and Crime 2021); however, many analogues eventually become controlled due to their unknown toxicologic and pharmacological effects (Pasin et al. 2017b). This cat-and-mouse game between law enforcement and clandestine laboratories has led to the proliferation of many new analogues and is a cause for significant concern for analytical laboratories.

As of December 2020, more than 1,000 different NPS have been reported to the UNODC Early Warning Advisory (EWA) from 126 different countries. The NPS market is primarily dominated by stimulants, such as synthetic cathinones, aminoindanes, and piperazines, and synthetic cannabinoids, accounting for 36% and 29% of the market, respectively. Classic hallucinogens, predominantly hallucinogenic phenethylamines, account for 15% of the market, while opioids represent 9% (United Nations Office on Drugs and Crime 2021), showing a large increase from the 2% of NPS reported in 2014 (United Nations Office on Drugs and Crime 2020). Synthetic opioids are a class of NPS of particular concern for toxicologists, due to the significantly higher potency of these compounds when compared to their traditional counterparts (Suzuki and El-Haddad 2017; Zawilska 2017). The increasing proliferation of these NPS, in addition to the often-considerable delay between the early use of a new compound and the availability of relevant certified reference materials (CRMs), demonstrates the inadequacies of traditional targeted screening and necessity of developing new, non-targeted screening strategies.

In recent years, there has been a continual push towards the use of high-resolution mass spectrometry (HRMS) techniques for the analysis of NPS. One of the main driving forces behind this trend is the ability of HRMS instruments to operate in a data-independent acquisition (DIA) mode (Pasin et al. 2017b). This method of acquisition allows for all precursor ions to be subjected to collision-induced dissociation (CID), which provides full scan tandem MS (MS/MS) data (Klingberg et al. 2021a; Noble et al. 2017). The major benefit of this acquisition mode is that data can be reviewed retrospectively for new compounds of interest, without the need for sample re-extraction and re-analysis (Pasin et al. 2017b).

Concurrent with the development of DIA, the expansion of metabolomics offers opportunities to improve non-targeted screening strategies (Courant et al. 2014; Fiehn et al. 2015; Szeremeta et al. 2021). Metabolomics aims to measure small changes within a multicellular system, in response to a given stimulus (Fiehn et al. 2015; Steuer et al. 2019; Szeremeta et al. 2021). The metabolome is described as a group of endogenous metabolites which are produced by a biological system (Junot et al. 2014). The inclusion of products metabolised from exogenous sources as either phase I or II metabolites is referred to as the xenometabolome (Courant et al. 2014; Steuer et al. 2019; Szeremeta et al. 2021). It is this unrestricted approach to sample analysis that makes the use of metabolomics so desirable.

The increase in the volume of data that is available to analysts, however, has necessitated more focus on ‘back-end’ data processing techniques to draw meaningful conclusions. With the availability of cheaper and more powerful computational processing (Margagliotti and Bollé 2019), statistical approaches, such as machine learning (Klingberg et al. 2021b; Meshref et al. 2020; Stanstrup et al. 2015) and molecular networking (Allard et al. 2019; Vincenti et al. 2020), have become more viable for toxicological applications. Machine learning algorithms enable a computer to ‘learn’ information directly from a data set without needing a predetermined equation to use as a model (Klingberg et al. 2021b). Molecular networking, on the other hand, is a technique that is capable of representing MS/MS data in a graphical form (Allard et al. 2019). This approach is able to identify potential similarities among all MS/MS spectra within a given dataset and correlate unknown but related molecules (Vincenti et al. 2020).

This review aims to provide an overview of the status of NPS analysis in toxicology. It will focus on the different techniques which have been used for both sample preparation and instrumental analysis. In addition, this review will highlight emerging areas of development, including the use of metabolomics-based techniques for drug screening, and novel data analysis methods applied to toxicological applications. Particular focus was given to advancements made since the review by Pasin et al. (2017b); however, topics that were not previously covered but published before 2017 are also reviewed here including novel data analysis and interpretation methods.

## Sample preparation and extraction

A particular challenge that can be associated with the analysis of NPS is the diverse range of analytes and the complex matrices in which they are often found. Therefore, appropriate sample preparation can be vital for successful analysis. Analytes are often found in urine as metabolised conjugates, requiring hydrolysis to liberate the free drug. The development of ‘dilute and shoot’ methods can alleviate this requirement (Borden et al. 2020). Some drug conjugates have been shown to be responsive to MS analysis (Dickerson et al. 2012), though they can often show poor sensitivity and lack commercially available standards for confirmatory analysis. Traditionally, hydrolysis was achieved through either acidic conditions or use of an enzyme, such as commercially available glucuronidase enzymes (Borden et al. 2020). Regardless of how hydrolysis is performed, once the free drug is liberated, it must be extracted from the matrix to facilitate analysis.

The two most common extraction procedures are solid-phase extraction (SPE) and liquid–liquid extraction (LLE). While these techniques are still widely used for toxicological analyses, they do have some drawbacks, including the use of large volumes of organic solvents (LLE) and being relatively time-consuming procedures (Borden et al. 2020). From a metabolomic perspective, these techniques also introduce bias as they limit the analytes to a targeted group, such as acidic or basic drugs. More recently, there has been a focus on the concept of ‘green chemistry’, which aims to reduce the environmental impact of chemical procedures, giving rise to the popularity of microextraction procedures. Both solid-phase microextraction (SPME) and liquid-phase microextraction (LPME) have been extensively reviewed and demonstrated applicability to toxicological analyses (Borden et al. 2020; He and Raynie 2017; Płotka-Wasylka et al. 2015). The key advantages of some emerging sample preparation methods are summarised in Table 1.

**Table 1:** Key advantages of emerging samples preparation methods

Technique	Key Advantages	Reference
Pressurised Liquid Extraction (PLE)	Increased extraction efficiency Potential for automation Lower solvent volumes required Faster extraction times Multi-analyte extraction	Carabias-Martínez et al. (2005); Montesano et al. (2017)
Dispersive Liquid–Liquid Microextraction (dLLME)	Smaller solvent and sample volumes than traditional LLE Greater enrichment factor Cleaner extracts Lower matrix effects Faster extraction times Multi-analyte extraction	Vincenti et al. (2019)
Quick, Easy, Cheap, Effective, Rugged, and Safe Extraction (QuEChERS)	Simple, two-step process Faster extraction times Increased sensitivity of detection	Anastassiades et al. (2003); Hasegawa et al. (2018)

In addition to microextraction techniques, the use of pressurised liquid extraction (PLE) as an effective multi-class extraction procedure for NPS from hair samples has been explored. PLE uses solvents at high temperatures and pressures, without their critical point being reached, in order to extract analytes from solid or semi-solid matrices (Montesano et al. 2017). Montesano et al. (2017) demonstrated the applicability of a PLE technique for the multi-class extraction of NPS from hair samples. The authors noted that the traditional extraction procedure used for hair samples involved NaOH matrix dissolution, followed by either SPE or LLE clean-up, which can be unsuitable for some drugs. As such, a PLE method was developed which demonstrated efficacy for the extraction of synthetic cannabinoids, cathinones, phenethylamines and piperazines. The collected hair samples were extracted using a 70:30 (*v/v*) water–methanol mix at a pressure of 100 bar and a temperature of 120 °C. The extract was then centrifuged, and the supernatant further extracted by SPE. The method was validated with matrix effects found to be less than 15% for all analytes (Montesano et al. 2017).

Vincenti et al. (2019) investigated the use of dispersive liquid–liquid microextraction (dLLME) in addition to PLE to extract a range of drugs of abuse from hair samples, including both NPS and traditional drugs of abuse. In this study, a mixture of 0.15 M formate buffer (pH 3.5) and isopropanol (80:20 *v/v*) was used for extraction at 100 bar and 150 °C. Following extraction and centrifugation, a dLLME procedure was carried out using isopropanol as a dispersing solvent and chloroform as an extraction solvent. In order to determine the effectiveness of the PLE-dLLME extraction procedure, the samples were compared to hair samples extracted using an alkaline digestion or a simple methanol extraction. It was found that analyte recoveries were generally lower than the other techniques, especially methanolic extraction; however, the extracts were a lot cleaner and less matrix effects were observed (Vincenti et al. 2019).

In addition to PLE, the quick, easy, cheap, effective, rugged, and safe (QuEChERS) extraction method has started seeing use in toxicological analyses. This method, first presented by Anastassiades et al. (2003), involves a simple, two-step extraction process including acetonitrile in the presence of a salt, commonly magnesium sulfate or sodium chloride, for extraction and dispersive SPE for clean-up (Yang et al. 2020). While QuEChERS has traditionally been used for the analysis of pesticides in food products, some recent studies have explored its application to more toxicologically relevant drug analyses. Hasegawa et al. (2018) employed QuEChERS for the identification and quantification of synthetic cannabinoid metabolites in human urine. Two different QuEChERS cartridges were used, both of which contained magnesium sulfate and end-capped octadecylsilane; however, one cartridge contained *N*-propylethylenediamine as a primary secondary amine (PSA) and the other did not. Acetonitrile was added to the QuEChERS tubes with the urine samples in a 9:1 ratio. All the analytes were identified using both extraction methods, however the presence of the PSA increased the sensitivity of detection, showing a 15-fold increase in peak areas (Hasegawa et al. 2018).

While matrices such as whole blood, plasma, serum, and urine are commonly used for toxicological analyses, and are considered the ‘conventional’ matrices, there has been significant research into the use of alternate matrices for drug screening. Oral fluid and hair have become increasing popular as matrices of interest in toxicology for different applications. Oral fluid has been considered a direct filtering of the blood as the salivary glands are highly perfused with blood. Much of the interest in this matrix is due to the simplified sample collection, which is quicker, easier, and non-invasive, in comparison to matrices such as blood and urine. On the other hand, this matrix can be variable, being influenced by factors such as circadian rhythm, age, gender, and health, among others. From an analytical perspective, the sample volume is generally limited, and analytes may be present in low amounts, requiring highly sensitive detection methods (de Campos et al. 2021). Additionally, hair analysis can be used to detect the presence of drugs and some metabolites. While it is still unclear exactly how drugs are incorporated into the hair, it is generally accepted that it involves passive diffusion through blood capillaries to the base of the hair follicle (Mantinieks et al. 2018). Similar to oral fluid, the collection of hair samples is easy and non-invasive and can help provide a timeline of drug use through segmental analysis; however, the detection of recent drug use (within approximately 7 days) is not possible (de Campos et al. 2021).

In addition to hair and oral fluid, a number of other unconventional matrices have been reviewed in the literature, including sweat and vitreous humor (de Campos et al. 2021), bile (Bévalot et al. 2016), nails (Solimini et al. 2017), bone marrow (Cartiser et al. 2011), and cerumen (ear wax) (Meier et al. 2017). The use of human breast milk and meconium, the first stool excreted by a newborn, has also been investigated in order to identify licit and illicit drug use by mothers during pregnancy (de Campos et al. 2021). While each of these matrices have unique advantages and disadvantages, it is important for an analyst to select the most appropriate matrix for the case under investigation and alternative matrices can be useful in situations where blood and/or urine is not available.

## NPS screening—instrumental analysis

Numerous analytical techniques have been developed to exploit the advantages of HRMS for the screening and confirmatory analysis of NPS. While most of these techniques involve the use of a coupled separation instrument, most commonly liquid chromatography (LC) or gas chromatography (GC), there have been some direct HRMS analyses reported in the literature, leveraging techniques such as matrix-assisted laser desorption/ionisation (MALDI) and paper spray ionisation.

The most common NPS screening methods involve the use of LC separation coupled to a variety of HRMS platforms, frequently QTOF or Orbitrap instruments. The major driving force behind the preferential use of LC over GC is that analytes of interest do not need to be volatile and derivatisation is not required (Pasin et al. 2017b). Many different methods optimised for a range of different chemical classes have been reported in the literature. Table 2 summarises the LC-HRMS-based screening methods recently presented in the literature.

**Table 2:** Summary of newly presented LC–MS-based screening methods

Drug classes monitored	Instrument	Analysis mode	Analyte identification	Reference
Broad spectrum	Orbitrap	Full scan MS	Accurate mass and RT alignment to standards	Mokhtar et al. (2020)
Broad spectrum	Orbitrap	Full scan MS	Accurate mass and RT alignment to standards	Stephanson et al. (2017)
Designer benzodiazepines	Orbitrap	Full scan MS (Screening) Parallel Reaction Monitoring (Confirmation)	Accurate mass and RT alignment to standards	Pettersson Bergstrand et al. (2018)
Synthetic opioids	QTOF	Data-dependant acquisition with inclusion list	Mass spectral library search	Krajewski et al. (2020)
Synthetic opioids	QTOF	Data-independent acquisition	Mass spectral library search (targeted) Monitoring of class-specific cleavages (non-targeted)	Noble et al. (2017)
Designer benzodiazepines	QTOF	Data-independent acquisition	Mass spectral library search (targeted) Common fragmentation pathways and in silico fragmentation (non-targeted)	Mollerup et al. (2017)
Synthetic opioids	QTOF	Sequential Windowed Acquisition of All Theoretical Fragment Ion Mass Spectra	Mass spectral library search	Salomone et al. (2021)

While the use of multi-reaction monitoring (MRM) and MS^1^ monitoring of pseudomolecular ions can be useful for drug screening, they do not provide a great deal of structural information or allow for the detection of unknown compounds. Non-targeted analyses are becoming increasingly popular due to the ever-changing nature of the NPS market. To this end, Krajewski et al. (2020) reported a method for the detection of newly emerging synthetic opioids using a QTOF instrument operated in data-dependent acquisition (DDA) mode. While this approach can allow for the detection of unknown compounds within a sample, there is still the possibility of analytes of interest being missed, especially when they have low abundance in comparison to the matrix. On the other hand, use of a DIA approach allows for both targeted screening in comparison to a library database as well as non-targeted screening using further data analysis and interpretation techniques. Another approach to DIA analysis is known as variable DIA, where the full mass-to-charge (*m/z*) range is broken down into smaller windows and analysed sequentially (Oberacher and Arnhard 2016). This can help reduce the noise present in extracted ion chromatograms (EICs) of product ions, as fewer precursor ions are being fragmented in each mass window. Salomone et al. (2021) demonstrated the use of Sequential Windowed Acquisition of All Theoretical Fragment Ion Mass Spectra (SWATH™) for the analysis of fentanyl analogues and metabolites in hair, using a total of 12 windows covering a mass range of *m/z* 230 to 450.

While the use of GC-HRMS for drug screening is not as common as LC-based techniques, Shevyrin et al. (2016; 2015) published multiple studies detailing the detection and characterisation of novel synthetic cannabinoids using both GC and LC-HRMS. In situations where CRMs and spectral data are not available for newly emerging compounds, GC-HRMS can present a useful orthogonal technique to LC-HRMS to assist with structural confirmation.

In addition to ‘conventional’ separation techniques, Gottardo et al. (2021) presented a capillary electrophoresis (CE)-HRMS method for the detection of a range of cathinones, phenethylamines and tryptamines in urine. While the use of CE is not commonplace within forensic toxicology, it can provide a useful alternate separation mechanism, which has demonstrated high separation efficiency, short analysis times and low sample volume requirements. Additionally, since CE techniques do not use a packed column, it can quickly change between different analytical conditions, without needing lengthy washing and equilibration times. On the other hand, CE can often have lower sensitivity than LC-based techniques and can demonstrate instability in migration times (Gottardo et al. 2021). Nevertheless, it can provide a useful orthogonal technique to LC-HRMS analysis.

While chromatographic or CE separation can help reduce noise in complex matrices, direct sample analysis techniques can also be used for rapid analysis. Due to the lack of chromatographic separation, these direct analysis techniques are commonly applied to seized samples, where the background matrix is less likely to affect the analysis. Birk et al. (2020) reported a method for the direct detection of novel phenethylamines, including 2C and NBOMe compounds, on street drug blotter samples using a paper spray ionisation QTOF-MS technique. Paper spray ionisation uses a combination of solvent and high voltages to create an ionised spray in a similar mechanism to electrospray ionisation. Using this approach, five different NPS compounds were able to be detected on street blotter samples with no complex sample preparation steps required. Similarly, Vandergrift et al. (2018) employed a paper spray ionisation approach to the detection of fentanyl and norfentanyl that had been spiked into diluted urine and commercially available analgesics, to simulate fentanyl being added to a street drug. It was found that by simply spotting a diluted urine sample or a methanolic suspension of a powdered sample onto a paper strip, fentanyl could be detected with limits of detection of 0.27 ng/mL and 0.66 ng/mL for urine and powder suspension, respectively.

Joye et al. (2020) reported the use of a MALDI-Orbitrap instrument for the detection of various novel phenethylamine and tryptamine compounds, alongside traditional drugs of abuse. Very little sample preparation was required, simply involving the dilution of the sample solution in a 1:1 ratio with a MALDI matrix solution before being spotted onto a stainless-steel plate for analyses. The authors found the results obtained by analysis using the MALDI technique comparable to conventional LC- and GC–MS analyses, suggesting that this approach could provide a viable alternative for rapid, high-throughput screening (Joye et al. 2020).

While most direct MS analysis techniques focus on screening for drugs prior to ingestion to reduce the potential effects from complex biological matrices, Usui et al. (2018) presented a probe electrospray ionisation (PESI) method designed for the direct detection of MT-45 in human tissue. Various tissue samples were placed on dedicated plastic sample plates before being placed into the PESI source. When the probe needle was brought into contact with the sample, a high voltage was applied to generate ions which could be introduced into the HRMS detector. The PESI-HRMS method presented was able to detect the presence of MT-45 in all tissue samples analysed, along with two potential hydroxylated metabolites with similar concentrations to conventional analyses (Usui et al. 2018). While the nature of direct analysis techniques makes it difficult to separate compounds with the same precursor mass, due to the lack of a chromatographic separation step, the presented direct analysis methods demonstrate the potential for such techniques to be used as alternate rapid screening analysis approaches.

## Wastewater-based epidemiology (WBE)

Understanding the usage patterns of NPS can be important not only for healthcare professionals and toxicologists to assess the risks posed by particular compounds, but also for policymakers to make informed decisions with regards to law enforcement activities (Bijlsma et al. 2019). Estimating the prevalence of NPS within the community can be quite challenging, however, as information obtained from drug seizures, forensic analyses and medical reports can often be outdated and not representative of the constantly shifting market (Salgueiro-González et al. 2019). Similarly, surveys of the population can be biased due to the users’ limited knowledge of the substances they are taking. It is difficult for a single measure to adequately provide the full picture of drug use within the community, and, therefore, it is common practice for a multi-indicator approach to be adopted (Archer et al. 2014). The use of urban wastewater and pooled urine samples has seen recent advances in providing anonymised but comprehensive information about the prevalence of NPS within the community (Bijlsma et al. 2021). This approach relies on the fact that traces of everything humans consume are excreted, whether unaltered or as metabolites. Therefore, monitoring biomarkers for relevant analytes of interest can be used to estimate drug use within a given population (Bijlsma et al. 2019).

Numerous studies have been conducted worldwide to investigate the prevalence of NPS use through screening of wastewater samples, which are summarised in Table 3. While wastewater analysis can be useful for broad-spectrum screening of NPS use within the community, there is also scope for its use in monitoring particular compounds, or classes, of concern. Additionally, studies have demonstrated the potential of retrospective analysis for screening historical samples, provided that suitable HRMS data were collected during the original analyses (Campos-Mañas et al. 2019).

**Table 3:** Summary of wastewater-based epidemiology studies worldwide

Location studied	Drug classes monitored	Extraction method	Analysis method	Drugs observed	Reference
Athens (Greece)	Broad spectrum	Four-layer SPE with acidic and basic fractions	Data-independent acquisition with reference to a mass spectral library and RT prediction model	24 targeted compounds, including synthetic cannabinoids, cathinones and opioids	Diamanti et al. (2019)
16 European Countries	Broad spectrum	Two different SPE cartridges	Full Scan MS and data-dependant MS^2^ with reference to a mass spectral library	13 different NPS, mostly phenethylamines	Salgueiro-González et al. (2019)
Minnesota (USA)	Opioids	Retrospective analysis of samples previously extracted through SPE	Retrospective screening of previously analysed samples and additional data-dependant MS^2^ reanalysis of sample with reference to a mass spectral library	10 opioids identified, 6 more putatively identified. Tramadol and dextromethorphan most common	Campos-Mañas et al. (2019)
Australia-wide	Cannabinoids	Both LLE and SPE	Multiple reaction monitoring	THC-COOH detected at all sites and CBD identified at 8 sites. Three synthetic cannabinoids also detected	Pandopulos et al. (2020)
South Australia	Prescribed and designer benzodiazepines	Vacuum filtration and SPE	Multiple reaction monitoring	Ten different analytes, with oxazepam most prevalent. Two compounds not marketed in Australia detected, indicating illicit use	Bade et al. (2020)

Pandopulos et al. (2020) noted that the extraction of cannabinoids from wastewater matrices can be particularly challenging due to their hydrophobic nature. To this end, an alternate method was developed for the analysis of sewage sludge and biosolids, as they can capture the nonaqueous components of wastewater and have been shown to contain the bulk of phytocannabinoids (Pandopulos et al. 2021). In this study, the sludge samples were filtered under vacuum to remove as much of the aqueous fraction as possible, before applying an LLE method. The analytical method used a QTrap instrument operated in MRM mode. The developed method was then applied to sludge samples collected from a major municipal treatment plant in Australia. The analyses showed three cannabinoids, namely Δ^9^-tetrahydrocannabinol (THC), 11-nor-9-carboxy-Δ^9^-tetrahydrocannabinol (THC-COOH) and cannabidiol (CBD), at high concentrations, with THC being the most abundant analyte, found at concentrations up to 3200 µg/kg in raw primary sludge. This study demonstrates that, while wastewater analysis is effective for the identification of illicit drug biomarkers, targeting the solid fraction could help in the accurate estimation of the prevalence of lipophilic compounds, such as cannabinoids, in the community (Pandopulos et al. 2021).

In addition to wastewater analysis, pooled anonymous urine analysis has been conducted to help form a more accurate picture of NPS use within the community. Pooled urine samples can form a particularly useful adjunct to wastewater analysis as it can eliminate some of the potential complications, such as the unknown bacterial metabolism and stability of targeted analytes in wastewater (Archer et al. 2014). Archer et al. (2014) demonstrated the potential of such a technique to study the trends in the use of classical drugs of abuse and NPS by analysing pooled anonymous urine samples from portable urinals around London (UK) over a 6-month period. The pooled samples underwent enzyme hydrolysis before three different SPE techniques were applied to collect extracts for three distinct assays: a basic drug, general drug, and a synthetic cannabinoid screen. All extracts were analysed on an Orbitrap instrument operated using full scan MS and a selection of C_18_ columns for the different screens. This study found a total of 13 different NPS from a range of chemical classes present in the pooled urine samples, with mephedrone being the most consistently identified analyte. Interestingly, synthetic cannabinoids were not detected in any of the pooled urine samples; however, it was noted by the authors that degradation may have occurred from the samples pooling for 12-h prior to collection (Archer et al. 2014).

Following on from this work, Archer et al. (2020) later conducted a long-term monitoring study of pooled urine samples over a 5.5-year period. For this study, samples were prepared in a similar manner to their previous work; however, only the general drug and synthetic cannabinoid screens were applied. Samples were analysed using a ThermoFisher Scientific Q Exactive orbitrap instrument operating with full scan MS and both DDA and *All Ion Fragmentation* MS/MS modes using T3 and C_18_ columns for the general drug and synthetic cannabinoid screens, respectively. A total of 44 NPS were detected over the study period, with cathinones and synthetic cannabinoids being the most prevalent classes. By monitoring the long-term presence of NPS within pooled urine samples, it is possible to gauge the impacts of legislative changes, with the prevalence of cathinones decreasing following the introduction of the Psychoactive Substances Act in the UK, mirrored by a rise in the prevalence of other NPS (Archer et al. 2020). Both these studies demonstrate the potential of pooled urine as an additional matrix to study the NPS phenomenon and provide valuable information to relevant stakeholders.

## Data analysis and machine learning

The large volume of data collected by HRMS analyses necessitates the development of novel data analysis strategies to extract useful intelligence out of the veritable haystack of information. These strategies can range from relatively simple processes, such as generating EICs for common product ions, to more advanced, computational-based techniques, such as machine learning and molecular networking.

### Suspect screening databases and mass spectral libraries

Given the continual proliferation of NPS and the constant introduction of new compounds, maintaining an up-to-date suite of reference materials is impractical and unrealistic for a toxicological laboratory. While there are a number of freely available MS libraries and data repositories, such as MassBank (Horai et al. 2010), mzCloud (HighChem LLC 2021), RESPONSE (https://www.policija.si/apps/nfl_response_web/seznam.php) and the NPS Data Hub (https://nps-datahub.com/) (Urbas et al. 2018), the scope of NPS covered by these resources varies and the databases themselves can be difficult to incorporate into screening processes across different vendor platforms (Mardal et al. 2019). In response to these challenges, Mardal et al. (2019) presented an online, crowd-sourced NPS database named HighResNPS. This database allows users to submit product ion data from their analysis of NPS and provides consensus data on diagnostic product ions based on their frequency of reporting. The database is also kept up to date through the addition of compounds reported by the EMCDDA, UNODC, Drug Enforcement Administration and other drug monitoring bodies. This database can be downloaded in the relevant vendor software formats to allow searching against the exact precursor masses and diagnostic product ions (Davidsen et al. 2020; Mardal et al. 2019).

The applicability of this crowd-sourced database to the analysis of seized materials was demonstrated by von Cüpper et al. (2020) who submitted five powder samples obtained from Danish authorities to HRMS analysis and conducted an accurate mass search including both precursor and product ions. In all five cases, the combination of accurate mass and fragmentation information provided by HighResNPS allowed for the tentative identification of the NPS present (von Cüpper et al. 2020). While the structural elucidation of isomeric compounds remains challenging, owing to the identical fragmentation patterns, tools such as these can be invaluable in providing preliminary information regarding the presence of novel analogues in analysed samples.

### Product ion searching

While mass spectral databases are a valuable tool for use in screening assays, they still leave the potential for false negative results where no previous data is available. As such, the development of non-targeted analysis methods which do not rely on these databases or CRMs is essential for comprehensive screening of toxicological samples. One such method which has been developed for this purpose is the use of diagnostic product ions to monitor specific classes of compounds, rather than individual analytes. It has been found previously that different HRMS instruments generally present comparable fragmentation patterns following CID (Mardal et al. 2019), which is crucial for these screening approaches to work.

There have been numerous studies that have investigated the CID pathways of various classes of NPS. Fornal (2014) studied the fragmentation of substituted cathinones and discovered that most known cathinones can be separated into nine distinct classes. These classes could be differentiated by their double bond equivalent (DBE) and the characteristics of the amine group present in the structure. From their study, it was found that the different subclasses of cathinones follow five generic fragmentation pathways and produce several diagnostic product ions which may be viable for non-targeted screening (Fornal 2014). Additionally, Fornal found that many protonated cathinones produce odd-electron product ions following CID, which contradicts the even-electron rule of fragmentation (Fornal 2013). This means that inclusion of odd-electron product ions into screening methods may be essential for the detection of novel cathinone analogues.

Pasin et al. (2017a) explored the CID pathways of hallucinogenic phenethylamines, namely 2,5-dimethoxyphenethylamines (2C-X), 2,5-dimethoxyamphetamines (DOX), and N-(2-methoxybenzyl) (25X-NBOMe) compounds. These compound classes showed several common neutral losses and diagnostic product ions which could be exploited for non-targeted screening strategies. The applicability of these findings to toxicological screening was then demonstrated using EICs and neutral loss filtering (NLF). It was found that the presence of a compound belonging to either the 2C-X or DOX groups can be detected by monitoring their common neutral losses, although they cannot be differentiated by NLF alone. Furthermore, the generation of EICs for diagnostic product ions allowed for the detection of relevant compounds in the samples analysed and could differentiate between analytes belonging to each class (Pasin et al. 2017a).

Noble et al. (2017) investigated the application of product ion searching (PIS) techniques to fentanyl analogues in conjunction with a DIA screening method. The authors found that a characteristic, class-specific cleavage of the C-N bond between the piperidine ring and amide moiety was present for all 50 fentanyl analogues analysed and could be used to screen for novel 4-anilidopiperidine fentanyl analogues. This was supported by the retrospective analysis of 2,339 authentic whole blood samples to identify 56 fentanyl, 5 alfentanil and 1 remifentanil positives (Noble et al. 2017). These findings demonstrate the usefulness of such screening techniques for routine casework. Klingberg et al. (2019) expanded upon this work to include additional classes of novel synthetic opioids (NSOs), namely the AH- and U-series opioids, along with MT-45 and the W- series pseudo-opioids. Consistent with the findings of Noble et al*.*, this study showed that each of the subclasses presented several diagnostic product ions that could be exploited for inclusion in a non-targeted screening method (Klingberg et al. 2019). Several spiked samples were analysed in equine plasma, and it was found that these diagnostic product ions could be used to detect different opioids down to concentrations of 0.05 ng/mL (Klingberg et al. 2021a, 2019). All these studies demonstrate the efficacy of PIS approaches to a broad spectrum of NPS and their potential to detect novel analogues which have yet to be included in mass spectral databases or have no CRM available.

### Mass defect filtering (MDF) and Kendrick mass defect (KMD)

Mass defect filtering (MDF) is another technique with the potential to interrogate the vast amounts of data acquired by HRMS instruments. The mass defect of a compound is defined as the difference between the exact mass and nominal integer mass (Sleno 2012; Zhang et al. 2009). Compound classes often have similar mass defects, or demonstrate specific trends with increasing nominal mass (Sleno 2012). This technique was first reported in a toxicological context by Grabenauer et al. (2012), who investigated its use in the screening of herbal products for synthetic cannabinoids. The authors reported that the MDF workflow was able to detect a compound that was not originally present in the total ion chromatogram.

An extension of MDF, known as Kendrick mass defect (KMD), has also shown promise for application to the screening of toxicological samples. This altered mass scale allows for the identification of a group of compounds that differ by a specific repeating mass unit, such as a methylene group (–CH_2_) (Hughey et al. 2001; Sleno 2012). The applicability of this technique for toxicological screening was first demonstrated by Anstett et al. (2018), who investigated the use of KMD analysis for the screening of various phenethylamine classes; namely 2C-X, aminopropylbenzofuran and 25X-NBOMe compounds. The authors were able to define a KMD filter which successfully differentiated the 2C-X compounds from the other structurally similar classes analysed in the study (Anstett et al. 2018). This demonstrated the potential selectivity of KMD analysis when screening for specific chemical classes.

The use of KMD analysis was further evaluated by Klingberg et al. (2021a) who investigated its application to synthetic opioids. This utilised a custom-built program developed by Pasin (2018) in the Visual Basic for Applications environment for Microsoft Excel (named *DefectDetect*). Spiked samples of equine plasma, containing a representative panel of synthetic opioids, were analysed using DIA and six different KMD filters applied to cover a broad scope of synthetic opioids. The authors found this approach was able to reliably detect most of the synthetic opioids present down to a concentration of 0.1 ng/mL. This was possible without targeting the known precursor mass of spiked compounds. It was noted, however, that the structural diversity within the different subclasses of opioids presented a challenge, as analogues which incorporate significant structural changes may not be captured by the implemented KMD filters. While this presents a drawback for the application of these techniques to NPS classes that display significant structural diversity, the use of KMD analysis can be beneficial to assist with the non-targeted screening of toxicological samples. One of the key advantages of KMD analysis and PIS is that they can be applied alongside currently implemented screening workflows, regardless of the vendor software being used at a given laboratory (Klingberg et al. 2021a). From an operational perspective, this is a significant advantage as it does not require users to implement and validate a new method to leverage these data analysis techniques.

### Metabolomics-driven approaches for NPS detection

Metabolomics-driven approaches for NPS focus on identifying potential biomarkers, through variations in the metabolome post-administration of a drug, which allows for an alternative approach to current detection methods, therefore reducing limitations in the detection of NPS (Steuer et al. 2019).

There are two different approaches to metabolomics in toxicological screening: targeted and non-targeted. Targeted metabolomics measures a known number of endogenous compounds and exogenous metabolites (Szeremeta et al. 2021). The applications of targeted metabolomics are often limited, however, as it requires reference standards for these metabolites for method development, validation, and quality control purposes, which are often unavailable or cost-prohibitive (Bade et al. 2019; Steuer et al. 2019). With growing numbers of NPS being reported, this approach would also be perpetually outdated. On the other hand, non-targeted metabolomics, also known as metabolic fingerprinting, is not limited to a predefined list of metabolites and therefore aims to analyse everything detected in the sample (Narduzzi et al. 2020; Szeremeta et al. 2021).

Non-targeted metabolomics generates semi-quantitative data and generally requires retrospective analysis to confirm the identity of metabolites of interest (Steuer et al. 2019). For this purpose, MS/MS experiments are used to gather further information on the identified metabolites (Pan and Raftery 2007). The general workflow of data processing for metabolomics analysis is well described by Scalbert et al. (2009). These steps include data pre-processing, normalisation, statistical tests, and metabolite identification. Statistical analysis requires data pre-processing and normalisation to account for biological and analytical variations (Junot et al. 2014; Narduzzi et al. 2020). Two approaches to statistical analysis, supervised and unsupervised methods, are generally used in combination for the interpretation of metabolomics data. The most common unsupervised tool is principal component analysis (PCA), as it allows for reasonably simple visual inspection of the data. Alternatively, supervised methods are used for biomarker discovery, classification, and prediction. Partial least squares (PLS) and support vector machines (SVM) are the most frequently used supervised learning methods (Ren et al. 2015).

One of the most time-consuming processes of metabolomics is metabolite identification. Often MS/MS spectra are used to facilitate the identification process (Ibáñez et al. 2014). Additionally, informatic tools, such as XCMS (https://xcmsonline.scripps.edu) (Smith et al. 2006) and MetFrag (https://ipb-halle.github.io/MetFrag/) (Ruttkies et al. 2016), may be used to aid in the analysis of fragmentation data or review in silico MS/MS spectra (Junot et al. 2014). Putative metabolite identifications rely on using databases to gain information about suspected metabolites, such as MS/MS data and chemical parameters (Courant et al. 2014). Junot et al. (2014) highlights a range of different databases, such as Metlin (https://metlin.scripps.edu) (Smith et al. 2005), Human Metabolome Database (HMDB, https://hmdb.ca/) (Wishart et al. 2017), Kyoto Encyclopedia of Genes and Genomes (KEGG, https://www.genome.jp/kegg/) (Kanehisa et al. 2004) and PubChem (https://pubchem.ncbi.nlm.nih.gov/), (Wang et al. 2011), available to analysts to assist metabolite identification.

Metabolomics has applications in many fields including doping, drug discovery, health and disease monitoring, and forensic toxicology (Cawley and Keledjian 2017; Mollerup et al. 2019; Shao and Le 2019; Wang et al. 2020). Table 4 provides an overview of studies which demonstrate the applicability of metabolomic-based approaches to toxicological casework. In addition, Steuer et al. (2020) investigated the effects on the metabolome of three different drugs, namely MDMA, amphetamine and 4-methylmethcathinone (mephedrone). When using statistical analyses to compare the effects seen from administration of all the analytes of interest, it was found that two specific endogenous compounds, linoleic acid and pregnenolone sulfate, were significantly altered. An alternate approach for identifying metabolic pathways of interest was investigated to reduce the data bottleneck that comes with non-targeted data analysis. This approach revealed that the aminoacyl-tRNA biosynthesis and linoleic acid metabolism pathways were significantly altered in all three administration studies (Steuer et al. 2020). This synonymous result revealed that both approaches were a viable means of analysing metabolomics data.

**Table 4:** Summary of metabolomic-based approaches to toxicological casework

Drug monitored	Biomarkers targeted	Results observed	Reference
γ-hydroxybutyric acid (GHB)	GHB; GHB-glucuronide; γ-aminobutyric acid (GABA); and γ-butyrolactone (GBL)	GHB found to be most relevant target analyte with a 10 µg/mL cut-off in post-mortem urine established to differentiate exogenous GHB	Busardò et al. (2017)
γ-hydroxybutyric acid (GHB)	3,4-dihydroxybutyric acid; 2,4-dihydroxybutyric acid; and glycolic acid	GHB related acids useful biomarkers in serum and urine as they extend the detection window of GHB	Jarsiah et al. (2021)
MDMA	Acylcarnitines; adenosine; adenosine monophosphate; inosine; lysophospatidylcholine; S-adenosyl-L-homocysteine; theiomorpholine 3-carboxylate; and tryptophan	Energy metabolism identified as the major site for metabolic changes in response to MDMA administration. Retrospective data analysis of prior screening data can be used to identify potential biomarkers of analytes of interest	Nielsen et al. (2016)
Heroin	Tricarboxylic acid cycle	Tryptophan and 5-hydroxytrptamine shown to decrease in serum, and tryptophan and 5-hydroxyindoleacetate shown to increase in urine following heroin administration	Zheng et al. (2013)

Following on from the work conducted by Steuer et al., Keen et al. (2021) investigated butorphanol administration in equine athletes using a non-targeted mass spectrometric approach employing an LC-HRMS instrument. The developed workflow enabled an extended window of detection for metabolic variation within the horse through a list of biomarkers of exposure, allowing for the effects of the doping to be identified long after the parent drug was below the limit of detection of the employed screening method. The implementation of these biomarkers in routine drug testing would, therefore, allow for improved doping control, especially when a significant delay between sample collection and the doping event occurs (Keen et al. 2021).

The applicability of metabolomics is ever growing due to its increased popularity within different scientific fields. It is likely that this will also be the case for forensic toxicological screening.

### Machine learning

The paradigm shift towards the use of HRMS for toxicological analyses has meant that there is a large volume of data available to analysts. Simultaneously, technological advances have meant that more powerful computational processing is widely available and cheaper, making the use of artificial intelligence approaches, such as machine learning, more realistic (Margagliotti and Bollé 2019). Mitchell (1997) defined machine learning as, ‘a computer is said to learn from experience with respect to some task and some performance measure, if its performance on said task improves with experience’. There are two distinct approaches to machine learning, namely supervised and unsupervised approaches. Supervised machine learning takes a known set of inputs (the training set) and known responses to those inputs (the output) and trains a model to predict responses for new input data. Supervised learning models can be further categorized as either ‘classification’ or ‘regression’ models. Classification models aim to predict a discrete output, such as a drug class, whereas regression models attempt to predict an outcome that falls within a continuous space, such as a time range in the case of retention time (RT) prediction. Alternatively, unsupervised machine learning is useful when an analyst wishes to explore data without specific goals or previous knowledge of the information contained within the data (Margagliotti and Bollé 2019).

Machine learning approaches have been previously applied to the field of drug discovery, most notably though the investigation of structure–activity relationships (Ekins 2018; Luechtefeld et al. 2018). RT prediction has been extensively studied using regression models for the study of environmental contaminants (Aalizadeh et al. 2019; Pyke et al. 2019) and more recently for drug analysis (Klingberg et al. 2021b; Miller et al. 2013; Mollerup et al. 2018).

The use of supervised machine learning algorithms, such as artificial neural networks (ANNs), for probabilistic feature recognition in LC-HRMS data has been suggested. Woldegebriel and Derks (2017) theorised that the detection of all possible peak features within a given sample can be considered a pattern recognition problem; therefore, a technique such as ANN can be especially useful. Features of interest within both the LC and MS space have unique characteristics, such as peak shapes and *m/z* patterns, that an algorithm can be trained to recognise. The applicability of the developed ANN was examined using two data sets analysed across two different LC–MS systems and known to contain a range of xenobiotics and pesticides. The output of this algorithm provided a two-dimensional coordinate, including both RT and *m/z*, along with a posterior probability of whether these coordinates correspond to the centre of a peak feature. The authors noted that there was no correlation between the intensity of a signal and the probability of feature detection, indicating the ANN was generalising sufficiently and could identify all potential features within a sample (Woldegebriel and Derks 2017). The identified features could then be compared to libraries/databases or undergo further processing to achieve putative identification. This approach is comparable to molecular feature extraction (MFE) algorithms that can be found in some proprietary software, such as Agilent Technologies *MassHunter Profinder*. MFE algorithms attempt to locate individual sample components (often called molecular features) within complex chromatograms (Sana et al. 2008). Both approaches allow for the simplification of complex matrices into the different components that are present.

While feature detection is important for identifying an analyte of interest within a sample, high-throughput screening can benefit from a simple binary classification of a blank sample vs. a drug containing sample. Streun et al. (2020) demonstrated the potential of using ANNs for such a purpose. In this study, the authors applied an ANN approach to raw HRMS-DIA data (i.e. not extracted mass spectra) acquired using SWATH MS^2^ mode. The raw HRMS data underwent pre-processing using the R programming language, allowing the data to be structured for application to the ANN. Initially, the network was successfully trained to differentiate between blank and drug-containing solvent samples using three different software platforms, namely KNIME, Keras and a custom-built Python program. Following the proof-of-concept using the solvent samples, the authors applied the approach to a batch of more than 150 authentic blood samples, 59 of which were considered blank and the rest containing relevant xenobiotics, including cocaine, amphetamine, and zolpidem. The determined sensitivity and specificity of the trained ANNs were within a suitable range for routine laboratory use and the difference between the software platforms tested was marginal (Streun et al. 2020). While this approach does not allow for the identification of the analyte of interest, it does show significant potential for settings such as workplace drug testing, where the prevalence of negative samples is high.

Another potential application for machine learning approaches was presented by Guan et al. (2021). In this study, the authors employed the metabolomics-driven software, *Compound Discoverer* (Thermo Fisher Scientific 2021), to extract all compounds present in a group of 13 plasma samples, two of which had been spiked with relevant analytes. This extraction process, however, identified 8343 compounds within the dataset, making it very difficult for an analyst to manually search for analytes of interest. To address this, a mathematical model was developed consisting of two algorithms to calculate the ratio of the mean (ROM) and outlier index (OLI), enabling the identification of analytes among the extracted compounds. The applied model was able to successfully identify 55 of the 57 spiked drugs present in the samples. While the developed model was easy to use and incorporate into the *Compound Discoverer* processing workflow, it was noted that the success of this technique was reliant on the use of drug databases to identify the compounds (Guan et al. 2021).

Identification of novel NPS analogues, without relying on CRMs or comprehensive HRMS databases, can prove problematic (Pasin et al. 2017b). While there are vendor software packages available to assist with this process, such as *Compound Discoverer* (Thermo Fisher Scientific 2021) and *Molecular Structure Correlator* (Agilent Technologies 2011), understanding the general classification of an unknown compound can assist an analyst to affect a timely identification. Klingberg et al. (2021b) developed a prediction model to exploit the class-specific fragmentation that has been noted by previous studies (Klingberg et al. 2019; Noble et al. 2017) by using MS^2^ data to classify synthetic opioids as either fentanyl derivatives, AH- or U- series opioids. This proof-of-concept study found that a Naïve Bayes model provided the best outcome, with an overall accuracy of 89.5% and 100% classification accuracy for fentanyl derivatives. It was noted, however, that the model was limited by the number of compounds available in each subclass, and the increased prediction accuracy for the fentanyl derivatives could likely be due to their higher prevalence in the training and validation sets (Klingberg et al. 2021b). While this study focused on the prediction of synthetic opioid compounds, it demonstrates the potential for similar processes to be implemented where other NPS classes show class specific fragmentation patterns.

Many of these studies have applied machine learning to analytical data to detect features of interest or as a classification tool; however, machine learning has been applied to the structures of NPS themselves. Skinnider et al. (2021) reported the development of a deep generative model, termed DarkNPS, to predict novel structures using the known structures of NPS present on the HighResNPS database. The model was able to predict 176 of the 189 (93.1%) NPS-related compounds that were added to the database after the training set was finalised. The model also predicted (or sampled) some structures more often than others, allowing them to be ranked based on their sampling frequency, which may indicate how likely a predicted structure is to appear on the market.

#### Retention time prediction

Another aspect of machine learning that has recently been gaining popularity is RT prediction. While the use of mass spectrometric techniques to identify unknown compounds is clearly invaluable, the ‘front end’ chromatographic RT is quite often overlooked. Knowledge of a theoretical RT for a suspected compound can provide an analyst with evidence to help confirm a putative structure and, perhaps more importantly, eliminate false identifications. Traditionally, RT prediction has been studied for the detection and identification of a large variety of pesticides and environmental contaminants (Aalizadeh et al. 2019, 2016; Bade et al. 2015; Bride et al. 2021; Feng et al. 2021). More recently, however, the use of RT prediction modelling has been investigated for drug screening and anti-doping applications.

One of the main obstacles to the effective implementation of RT prediction in routine analysis is the use of unique chromatographic systems. This in turn means that the RTs predicted by one laboratory cannot be directly applicable to another and, therefore, specific prediction models are required. To mitigate this, Stanstrup et al. (2015) presented an online database, known as PredRet (http://predret.org), that allows for users to share RT information across laboratories and chromatographic systems. While many RT prediction approaches rely on the use of physicochemical properties for a given compound to model the quantitative structure–retention relationships (QSRR), PredRet generates predicted RT values by mapping the different RTs of known compounds between chromatographic systems. In this way, experimentally determined RTs of several compounds across two different chromatographic systems can be used to model the relationships between the two systems. If the RT of a given compound is known by one system, but not the other, the model can be used to predict the RT in the other system. While this approach cannot be used to compare RT values between very different chromatographic systems, such as hydrophilic interaction liquid chromatography (HILIC) and reversed-phase liquid chromatography (Stanstrup et al. 2015), it can still provide valuable information to an analyst when attempting to identify an unknown compound.

Barron and McEneff (2016) developed a generalised ANN model to predict the RTs of 1117 chemically diverse compounds in a range of complex matrices across ten different reversed-phase chromatographic systems. Unlike Stanstrup et al. (2015), however, the authors applied a more traditional approach to QSRR modelling, using 16 different molecular descriptors to generate predicted RTs. These molecular descriptors included unsaturation index, hydrophilic factor, Ghose–Crippen log*P* (AlogP), Moriguchi log*P* (MlogP), number of benzene-like rings, number of double and triple bonds, number of 4–9 membered rings, number of carbons, number of oxygens, p*K*_*a*_ (acid and base), log*P* and log*D*. Several different types of neural networks were also trialled, and it was determined that multilayered perceptrons provided the best predictions for 8 out of the 10 chromatographic systems. Following training of the ANNs, blind trials were conducted and an average predictive inaccuracy of 1.02 min for the blind trial compound was found across all the chromatographic systems. The authors, therefore, stated that RT prediction using ANNs, when reversed-phased chromatographic systems are used, is a viable method for implementation into non-targeted screening workflows (Barron and McEneff 2016).

The application of RT prediction modelling to anti-doping analysis was investigated by Miller et al. (2013). In this study, the authors used a dataset containing 86 different compounds included in the London 2012 Olympic and Paralympic Games drug testing schedule and developed an ANN prediction model using 18 different molecular descriptors. The molecular descriptors used in this study included the 16 used by Barron and McEneff (2016), as well as Alog*D* and Mlog*D* values. The authors first assessed the reproducibility of the chromatographically generated RTs to determine the viability of an RT prediction approach and found a maximum RT variability of 0.35 min across all samples. A feed-forward, back-propagated multilayered perceptron network with two hidden layers of five and four, respectively, provided the best prediction results and could predict 80 of the 86 compounds tested (~ 93%) within 0.5 min of their experimentally determined RTs. The study also investigated the contribution of each molecular descriptor to the predicted RT by sequentially removing one descriptor and calculating the percent change in the accuracy of the predicted RT values. From this investigation, it was found that the number of carbon atoms, number of double bonds, number of oxygen atoms, Alog*P* values and number of 9-memebered rings were the five most contributing descriptors to the overall accuracy of the model (Miller et al. 2013).

Following on from the work conducted by Miller et al. (2013), Talebi et al. (2015) investigated the use of PLS-related methods for selecting appropriate variables to be used for RT prediction modelling. The authors computed 825 different molecular descriptors for 86 suspected sports doping compounds to predict their RTs in a reversed-phase chromatographic system. Six different multivariate analysis methods were applied to select descriptors for RT prediction. It was found that all the models trained using a subset of descriptors selected by the multivariate methods outperformed the PLS model trained with all the descriptors. The number of descriptors that were selected by each approach ranged drastically, from 28 descriptors for competitive adaptive reweighted sampling (CARS) to 263 for Monte Carlo uninformative variable elimination (MC-UVE); however, the authors determined that the CARS approach provided the best compromise between the number of descriptors selected and the prediction accuracy. This approach was able to predict the RT of the compounds in the test set with prediction errors as low as 46 s and there was no evidence of any bias or systematic error in the predicted values, indicating the suitability of the model for the dataset used (Talebi et al. 2015).

Klingberg et al. (2021b) investigated the use of regression modelling to predict RT data for a range of synthetic opioid compounds. The authors employed 13 molecular features to evaluate four different regression models and found that the Gaussian Process Regression (GPR) model provided the best prediction accuracy for the dataset employed. Similarly to Miller et al. (2013), the authors investigated the effect of individual descriptors on the overall accuracy of the model. It was discovered that five of the descriptors led to a decrease in the prediction accuracy and were therefore, omitted from the optimised GPR model. Using the optimised model, it was found that 79.7% of the samples fell within ± 0.1 min of their experimentally determined RT, which indicates that the model would be suitable for application to non-targeted screening workflows. While this work focused on a subset of compounds relevant to toxicological screening, it demonstrates the viability of such an approach to broader applications. Unlike the works presented by Stanstrup et al. (2015) and Barron and McEneff (2016), this model was specific to the chromatographic system employed by the author, and, therefore, it would require retraining with chromatographic data specific to the system it is being applied to (Klingberg et al. 2021b).

Mollerup et al. (2018) took a broader approach to RT prediction, developing an ANN model for 869 different compounds using 105 molecular descriptors. The compounds included in this study covered a range of pharmaceuticals, drugs of abuse and their metabolites, making it highly relevant for toxicological screening. In addition to the RT prediction, the developed model incorporated ion mobility spectrometry (IMS) data to predict collision cross sections (CCS) of the included compounds. The CCS of a compound is related to its size, shape and charge (Mollerup et al. 2018) and has been shown to be matrix and system independent (Regueiro et al. 2016). This makes it a useful orthogonal technique to chromatographic separation and can help reduce the number of false positive identifications. In this study, the authors found that a four-layered multilayer perceptron ANN provided the best optimisation for the combined RT-CSS prediction model, with 91.9% of the compounds falling within ± 2 min for the RT prediction and 5% relative CCS error (Mollerup et al. 2018). While the RT prediction accuracy in this study is lower compared to other studies, it is important to note that this study included a broad range of chemically diverse compounds, and, therefore, it can be expected that some variation will be observed. Additionally, the inclusion of the CCS prediction to compliment the predicted RT values can also help affect putative identification of compounds.

More recently, Pasin et al. (2021) developed a single ANN-based RT prediction model capable of incorporating multiple chromatographic systems using a machine learning technique called ‘one-hot encoding’. The model was trained using data from HighResNPS and included laboratories that provided 50 analytes or more. The model was able to predict 81% and 97% of the test set (*n* = 193) within ± 1 and 2 min, respectively. The results also showed that a model incorporating all laboratories outperformed individually trained models, particularly when there were smaller numbers of compounds. The model is used to predict the RTs of all entries on HighResNPS for each laboratory included in the model to facilitate the development of personalised suspect screening libraries containing over 2000 NPS-related compounds (Pasin et al. 2021).

### Molecular networking

Molecular networking has been presented as a method to visualise complex, multi-dimensional HRMS data in a graphical form and was originally developed and validated for small molecules (Allard et al. 2019; Wang et al. 2016). This technique has been applied to the study of metabolomics, metabolite identification (van der Hooft et al. 2016), natural products research (Wang et al. 2016), drug discovery and precision medicine (Quinn et al. 2017). The molecular network itself provides a visual depiction of the spectra that are acquired in an MS^2^ experiment, which allows for the comparison of MS profiles. Within the network, each node represents a particular ion and its associated fragmentation spectrum. Links between the different nodes represent similarities in the obtained spectra (Allard et al. 2019; Vincenti et al. 2020). This means that a molecular network can provide both an overview of all the molecules which were detected and fragmented during a non-targeted MS experiment and their structural relationships (Allard et al. 2019). This approach, however, relies on the assumption that structurally related molecules will produce similar fragmentation patterns, in the same way that PIS does and, therefore, will be related within the molecular network (Quinn et al. 2017). If some compounds within a network can be identified and annotated, this structural information can then be propagated to unknown, but structurally similar, compounds allowing for dereplication within the sample (Wang et al. 2016; Yang et al. 2013). This information can be especially useful to an analyst for the identification of drug metabolites and novel NPS where analogues share a similar scaffold structure (Allard et al. 2019; Vincenti et al. 2020).

The application of this molecular networking approach to the context of forensic toxicology has been demonstrated by several studies. Allard et al. (2019) explored the potential uses of molecular networking combined with HRMS analysis of various biological matrices. This study utilised the Global Natural Products Social Molecular Networking (GNPS) online platform. GNPS is a data-driven platform which allows for the storage, analysis and dissemination of MS^2^ spectra (Aron et al. 2020; Wang et al. 2016). The analysis infrastructure of this online platform allows for automated generation of molecular networks (Wang et al. 2016). The authors first investigated the case of a lethal self-injection through the analysis of the contents of a syringe and a femoral blood sample taken from the victim. In this case, the main objective was to determine if any of the compounds present in the syringe were also found in the victim’s blood (Allard et al. 2019). It was discovered that only a single node contained a compound found in both the blood and syringe liquid. Further investigation allowed for the annotation of this node as the pesticide chlormequat, which was consistent with the official cause of death. In a second case, hair samples from the victim of a sexual assault were analysed. The hair was segmented into three sections, which correlated to the month before the incident, the month of the incident and the month after the incident, respectively. In this case, the objective was to determine if there were any suspected xenobiotics present only in the segment related to the month of the incident. Once again, inspection of the molecular network highlighted one node which was only found in the segment of interest, and correlated to the antihistamine doxylamine, which is commonly used in drug-facilitated sexual assault cases due to its sedative effects (Allard et al. 2019). Both cases demonstrate the effectiveness of a molecular networking approach to assist analysts to draw meaningful conclusions from complicated cases involving multiple samples and complex biological matrices.

Allard et al. (2019) also demonstrated the potential of a molecular networking approach for the exploration of NPS metabolism. This can be especially important as the metabolism of newly emerging NPS is often unknown. In this study, blood and urine samples of a patient who had taken the hallucinogenic 3-methoxyphencyclidine (3-MeO-PCP) were analysed and a molecular network generated. The node relating to 3-MeO-PCP was linked to a cluster of other molecules, indicating they possessed potentially similar chemical structures. Within this cluster, 12 previously described metabolites of 3-MeO-PCP were identified, along with five putative metabolites (Allard et al. 2019). More recently, Gicquel et al. (2021) identified several metabolites for a novel ketamine derivative, 2-fluoro-deschloroketamine (2F-DCK), using a molecular networking approach. In vitro experiments were conducted using both human liver microsomes (HLMs) and hepatic (HepaRG) cell line incubates to explore the metabolic process of this ketamine derivative. Additionally, post-mortem samples, including blood, bile, vitreous humor and urine, were taken from a self-intoxication fatality involving 2F-DCK. By using molecular networks to map the spectral data, 13 metabolites of 2F-DCK were identified from the in vitro studies, with a further seven metabolites identified in the post-mortem bile and urine samples (Gicquel et al. 2021). This approach could prove invaluable to toxicological analysis, especially in situations where the metabolic pathways of NPS are completely unknown.

Following on from the work completed by Allard et al., Yu et al. (2019) investigated the use of molecular networking for the classification of several cathinone and NBOMe derivatives. Nine NBOMe derivatives and eleven cathinone derivatives, as well as ephedrine and pseudoephedrine, were analysed and found to separate into two distinct clusters. Additionally, the authors analysed various spiked urine samples to determine the feasibility of using a molecular networking approach to screen for unknown NPS in biological samples. Nine NBOMe compounds, including two considered as unknowns (i.e. not included in the spectral database used) were identified in a single cluster (Yu et al. 2019). While this preliminary study may not account for all classes of NPS, especially where there is significant structural variation within a given class of NPS, it does demonstrate the potential of molecular networking as a useful tool to screen and categorise NPS.

In addition to the use of molecular networks for the screening of biological samples, Vincenti et al. (2020) demonstrated the potential of this approach to assist with the identification of compounds present within seized samples. In this study, a molecular networking approach was used to identify four different fentanyl derivatives within the seized samples, as well as two unexpected derivatives. These derivatives were not present in the mixed standards analysed alongside the seizures; however, they could be detected and putatively identified due to their connection to the cluster containing other fentanyl derivatives (Vincenti et al. 2020). All these studies demonstrate the potential for molecular networking approaches to assist analysts in the various facets of toxicological analyses.

## Conclusion

HRMS analysis is a powerful tool in the arsenal of toxicologists to combat the constant evolution of the NPS threat. Wastewater analyses have shown significant potential in their ability to model the overall consumption of drugs of abuse within a given community. While this may not have a significant impact on most day-to-day analyses, this intelligence can be invaluable in guiding healthcare professionals and policy makers in the implementation of harm reduction strategies. In addition, metabolomic approaches have been applied to drug screening. These approaches aim to characterise the effects that drugs of abuse have on the metabolome, which can provide new information that can be used for non-targeted analyses. Additionally, this approach can potentially identify new biomarkers that can be used to screen for drug use. With HRMS instruments becoming more commonplace in toxicological laboratories, the focus is shifting towards novel ways to use the large volume of data produced by these instruments. Many different data analysis and interpretation strategies have been developed to leverage the advantages of HRMS instruments and the increasing availability of powerful computational processing to analysts has made the prospect of machine learning assisted data processing more accessible. While there is still no single technique that can be used to provide reliable detection of all analytes of interest, the continual evolution of novel data interpretation methods is bringing that prospect closer to reality. In the meantime, the use of a ‘toolbox’ approach using multiple, complementary techniques can allow for the development of a rigorous analytical workflow to encompass a broad range of the chemical space.

## References

[CR1] Aalizadeh R, Thomaidis NS, Bletsou AA, Gago-Ferrero P (2016). Quantitative structure-retention relationship models to support nontarget high-resolution mass spectrometric screening of emerging contaminants in environmental samples. J Chem Inf Model.

[CR2] Aalizadeh R, Nika M-C, Thomaidis NS (2019). Development and application of retention time prediction models in the suspect and non-target screening of emerging contaminants. J Hazard Mater.

[CR3] Agilent Technologies (2011) Agilent MassHunter Molecular Structure Correlator (MSC) Software, United States of America G3335–90126. https://www.agilent.com/cs/library/usermanuals/public/G3335-90126_MSC_QuickStart.pdf

[CR4] Allard S, Allard P-M, Morel I, Gicquel T (2019). Application of a molecular networking approach for clinical and forensic toxicology exemplified in three cases involving 3-MeO-PCP, doxylamine, and chlormequat. Drug Test Anal.

[CR5] Anastassiades M, Lehotay SJ, Štajnbaher D, Schenck FJ (2003). Fast and easy multiresidue method employing acetonitrile extraction/partitioning and “dispersive solid-phase extraction” for the determination of pesticide residues in produce. J AOAC Int.

[CR6] Anstett A, Chu F, Alonso DE, Smith RW (2018). Characterization of 2C-phenethylamines using high-resolution mass spectrometry and Kendrick mass defect filters. Forensic Chem.

[CR7] Archer JRH, Dargan PI, Lee HMD, Hudson S, Wood DM (2014). Trend analysis of anonymised pooled urine from portable street urinals in central London identifies variation in the use of novel psychoactive substances. Clin Toxicol (phila).

[CR8] Archer JRH, Mendes F, Hudson S, Layne K, Dargan PI, Wood DM (2020). Evaluation of long-term detection trends of new psychoactive substances in pooled urine from city street portable urinals (London, UK). Br J Clin Pharmacol.

[CR9] Aron AT, Gentry EC, McPhail KL (2020). Reproducible molecular networking of untargeted mass spectrometry data using GNPS. Nat Protoc.

[CR10] Bade R, Bijlsma L, Miller TH, Barron LP, Sancho JV, Hernández F (2015). Suspect screening of large numbers of emerging contaminants in environmental waters using artificial neural networks for chromatographic retention time prediction and high resolution mass spectrometry data analysis. Sci Total Environ.

[CR11] Bade R, Tscharke BJ, White JM (2019). LC-HRMS suspect screening to show spatial patterns of New Psychoactive Substances use in Australia. Sci Total Environ.

[CR12] Bade R, Ghetia M, White JM, Gerber C (2020). Determination of prescribed and designer benzodiazepines and metabolites in influent wastewater. Anal Methods.

[CR13] Barron LP, McEneff GL (2016). Gradient liquid chromatographic retention time prediction for suspect screening applications: A critical assessment of a generalised artificial neural network-based approach across 10 multi-residue reversed-phase analytical methods. Talanta.

[CR14] Bévalot F, Cartiser N, Bottinelli C, Guitton J, Fanton L (2016). State of the art in bile analysis in forensic toxicology. Forensic Sci Int.

[CR15] Bijlsma L, Celma A, López FJ, Hernández F (2019). Monitoring new psychoactive substances use through wastewater analysis: current situation, challenges and limitations. Curr Opin Environ Sci Health.

[CR16] Bijlsma L, Bade R, Been F, Celma A, Castiglioni S (2021). Perspectives and challenges associated with the determination of new psychoactive substances in urine and wastewater-A tutorial. Anal Chim Acta.

[CR17] Birk L, de Oliveira SEF, Mafra G (2020). A low-voltage paper spray ionization QTOF-MS method for the qualitative analysis of NPS in street drug blotter samples. Forensic Toxicol.

[CR18] Borden SA, Palaty J, Termopoli V (2020). Mass spectrometry analysis of drugs of abuse: challenges and emerging strategies. Mass Spectrom Rev.

[CR19] Bride E, Heinisch S, Bonnefille B, Guillemain C, Margoum C (2021). Suspect screening of environmental contaminants by UHPLC-HRMS and transposable Quantitative Structure-Retention Relationship modelling. J Hazard Mater.

[CR20] Busardò FP, Kyriakou C, Marchei E, Pacifici R, Pedersen DS, Pichini S (2017). Ultra-high performance liquid chromatography tandem mass spectrometry (UHPLC–MS/MS) for determination of GHB, precursors and metabolites in different specimens: application to clinical and forensic cases. J Pharm Biomed Anal.

[CR21] Campos-Mañas MC, Ferrer I, Thurman EM, Sánchez Pérez JA, Agüera A (2019). Identification of opioids in surface and wastewaters by LC/QTOF-MS using retrospective data analysis. Sci Total Environ.

[CR22] Carabias-Martínez R, Rodríguez-Gonzalo E, Revilla-Ruiz P, Hernández-Méndez J (2005). Pressurized liquid extraction in the analysis of food and biological samples. J Chromatogr A.

[CR23] Cartiser N, Bévalot F, Fanton L, Gaillard Y, Guitton J (2011). State-of-the-art of bone marrow analysis in forensic toxicology: a review. Int J Legal Med.

[CR24] Cawley AT, Keledjian J (2017). Intelligence-based anti-doping from an equine biological passport. Drug Test Anal.

[CR25] Courant F, Antignac J-P, Dervilly-Pinel G, Le Bizec B (2014). Basics of mass spectrometry based metabolomics. Proteomics.

[CR26] Davidsen A, Mardal M, Linnet K, Dalsgaard PW (2020). How to perform spectrum-based LC-HR-MS screening for more than 1,000 NPS with HighResNPS consensus fragment ions. PLoS ONE.

[CR27] de Campos EG, da Costa BRB, dos Santos FS (2021). Alternative matrices in forensic toxicology: a critical review. Forensic Toxicol.

[CR28] Diamanti K, Aalizadeh R, Alygizakis N, Galani A, Mardal M, Thomaidis NS (2019). Wide-scope target and suspect screening methodologies to investigate the occurrence of new psychoactive substances in influent wastewater from Athens. Sci Total Environ.

[CR29] Dickerson JA, Laha TJ, Pagano MB, O'Donnell BR, Hoofnagle AN (2012). Improved detection of opioid use in chronic pain patients through monitoring of opioid glucuronides in urine. J Anal Toxicol.

[CR30] Ekins S (2018). Computational toxicology: risk assessment for chemicals.

[CR31] Feng C, Xu Q, Qiu X (2021). Evaluation and application of machine learning-based retention time prediction for suspect screening of pesticides and pesticide transformation products in LC-HRMS. Chemosphere.

[CR32] Fiehn O, Putri SP, Saito K, Salek RM, Creek DJ (2015). Metabolomics continues to expand: highlights from the 2015 metabolomics conference. Metabolomics.

[CR33] Fornal E (2013). Formation of odd-electron product ions in collision-induced fragmentation of electrospray-generated protonated cathinone derivatives: aryl α-primary amino ketones. Rapid Commun Mass Spectrom.

[CR34] Fornal E (2014). Study of collision-induced dissociation of electrospray-generated protonated cathinones. Drug Test Anal.

[CR35] Gicquel T, Pelletier R, Richeval C (2021). Metabolite elucidation of 2-fluoro-deschloroketamine (2F-DCK) using molecular networking across three complementary in vitro and in vivo models. Drug Test Anal.

[CR36] Gottardo R, Sorio D, Soldati G, Ballotari M, Porpiglia NM, Tagliaro F (2021). Optimization and validation of a new approach based on CE-HRMS for the screening analysis of novel psychoactive substances (cathinones, phenethylamines, and tryptamines) in urine. Electrophoresis.

[CR37] Grabenauer M, Krol WL, Wiley JL, Thomas BF (2012). Analysis of synthetic cannabinoids using high-resolution mass spectrometry and mass defect filtering: implications for nontargeted screening of designer drugs. Anal Chem.

[CR38] Guan F, You Y, Fay S, Li X, Robinson MA (2021). Novel algorithms for comprehensive untargeted detection of doping agents in biological samples. Anal Chem.

[CR39] Hasegawa K, Minakata K, Gonmori K (2018). Identification and quantification of predominant metabolites of synthetic cannabinoid MAB-CHMINACA in an authentic human urine specimen. Drug Test Anal.

[CR40] He Y, Raynie DE (2017). Microextraction and its application to forensic toxicology analysis. LC GC North America.

[CR41] HighChem LLC (2021) mzCloud—Advanced Mass Spectral Database. In. https://www.mzcloud.org/ Accessed 23rd June 2021

[CR42] Horai H, Arita M, Kanaya S (2010). MassBank: a public repository for sharing mass spectral data for life sciences. J Mass Spectrom.

[CR43] Hughey CA, Hendrickson CL, Rodgers RP, Marshall AG, Qian K (2001). Kendrick mass defect spectrum: a compact visual analysis for ultrahigh-resolution broadband mass spectra. Anal Chem.

[CR44] Ibáñez M, Sancho JV, Bijlsma L, van Nuijs ALN, Covaci A, Hernández F (2014). Comprehensive analytical strategies based on high-resolution time-of-flight mass spectrometry to identify new psychoactive substances. TrAC Trends Anal Chem.

[CR45] Jarsiah P, Roehrich J, Kueting T, Martz W, Hess C (2021). GHB related acids are useful in routine casework of suspected GHB intoxication cases. Forensic Sci Int.

[CR46] Joye T, Widmer C, Morger Mégevand R, Longère S, Augsburger M, Thomas A (2020). High-throughput qualitative and quantitative drug checking by MALDI HRMS. Front Chem.

[CR47] Junot C, Fenaille F, Colsch B, Bécher F (2014). High resolution mass spectrometry based techniques at the crossroads of metabolic pathways. Mass Spectrom Rev.

[CR48] Kanehisa M, Goto S, Kawashima S, Okuno Y, Hattori M (2004). The KEGG resource for deciphering the genome. Nucleic Acids Res.

[CR49] Keen B, Cawley A, Fouracre C, Pyke J, Fu S (2021). Towards an untargeted mass spectrometric approach for improved screening in equine antidoping. Drug Test Anal.

[CR50] Klingberg J, Cawley A, Shimmon R, Fu S (2019). Collision-induced dissociation studies of synthetic opioids for non-targeted analysis. Front Chem.

[CR51] Klingberg J, Cawley A, Shimmon R, Fouracre C, Pasin D, Fu S (2021). Finding the proverbial needle: non-targeted screening of synthetic opioids in equine plasma. Drug Test Anal.

[CR52] Klingberg J, Cawley A, Shimmon R, Fu S (2021). Towards compound identification of synthetic opioids in nontargeted screening using machine learning techniques. Drug Test Anal.

[CR53] Krajewski LC, Swanson KD, Bragg WA (2020). Application of the fentanyl analog screening kit toward the identification of emerging synthetic opioids in human plasma and urine by LC-QTOF. Toxicol Lett.

[CR54] Luechtefeld T, Rowlands C, Hartung T (2018). Big-data and machine learning to revamp computational toxicology and its use in risk assessment. Toxicol Res-UK.

[CR55] Mantinieks D, Gerostamoulos D, Wright P, Drummer O (2018). The effectiveness of decontamination procedures used in forensic hair analysis. Forensic Sci Med Pathol.

[CR56] Mardal M, Andreasen MF, Mollerup CB (2019). HighResNPS.com: an online crowd-sourced HR-MS database for suspect and non-targeted screening of new psychoactive substances. J Anal Toxicol.

[CR57] Margagliotti G, Bollé T (2019). Machine learning and forensic science. Forensic Sci Int.

[CR58] Meier SI, Koelzer SC, Schubert-Zsilavecz M, Toennes SW (2017). Analysis of drugs of abuse in Cerumen—correlation of postmortem analysis results with those for blood, urine and hair. Drug Test Anal.

[CR59] Meshref S, Li Y, Feng Y-L (2020). Prediction of liquid chromatographic retention time using quantitative structure-retention relationships to assist non-targeted identification of unknown metabolites of phthalates in human urine with high-resolution mass spectrometry. J Chromatogr A.

[CR60] Miller TH, Musenga A, Cowan DA, Barron LP (2013). Prediction of chromatographic retention time in high-resolution anti-doping screening data using artificial neural networks. Anal Chem.

[CR61] Mitchell TM (1997). Machine learning.

[CR62] Mokhtar SU, Kulsing C, Althakafy JT, Kotsos A, Drummer OH, Marriott PJ (2020). Simultaneous analysis of drugs in forensic cases by liquid chromatography–high-resolution orbitrap mass spectrometry. Chromatographia.

[CR63] Mollerup CB, Dalsgaard PW, Mardal M, Linnet K (2017). Targeted and non-targeted drug screening in whole blood by UHPLC-TOF-MS with data-independent acquisition. Drug Test Anal.

[CR64] Mollerup CB, Mardal M, Dalsgaard PW, Linnet K, Barron LP (2018). Prediction of collision cross section and retention time for broad scope screening in gradient reversed-phase liquid chromatography-ion mobility-high resolution accurate mass spectrometry. J Chromatogr A.

[CR65] Mollerup CB, Rasmussen BS, Johansen SS, Mardal M, Linnet K, Dalsgaard PW (2019). Retrospective analysis for valproate screening targets with liquid chromatography–high resolution mass spectrometry with positive electrospray ionization: an omics-based approach. Drug Test Anal.

[CR66] Montesano C, Vannutelli G, Massa M (2017). Multi-class analysis of new psychoactive substances and metabolites in hair by pressurized liquid extraction coupled to HPLC-HRMS. Drug Test Anal.

[CR67] Narduzzi L, Dervilly G, Audran M, Le Bizec B, Buisson C (2020). A role for metabolomics in the antidoping toolbox?. Drug Test Anal.

[CR68] Nielsen KL, Telving R, Andreasen MF, Hasselstrøm JB, Johannsen M (2016). A metabolomics study of retrospective forensic data from whole blood samples of humans exposed to 3,4-methylenedioxymethamphetamine: a new approach for identifying drug metabolites and changes in metabolism related to drug consumption. J Proteome Res.

[CR69] Noble C, Dalsgaard PW, Johansen SS, Linnet K (2017). Application of a screening method for fentanyl and its analogues using UHPLC-QTOF-MS with data-independent acquisition (DIA) in MSE mode and retrospective analysis of authentic forensic blood samples. Drug Test Anal.

[CR70] Oberacher H, Arnhard K (2016). Current status of non-targeted liquid chromatography-tandem mass spectrometry in forensic toxicology. TrAC Trends Anal Chem.

[CR71] Pan Z, Raftery D (2007). Comparing and combining NMR spectroscopy and mass spectrometry in metabolomics. Anal Bioanal Chem.

[CR72] Pandopulos AJ, Bade R, O'Brien JW (2020). Towards an efficient method for the extraction and analysis of cannabinoids in wastewater. Talanta.

[CR73] Pandopulos AJ, Simpson BS, Bade R (2021). A method and its application to determine the amount of cannabinoids in sewage sludge and biosolids. Environ Sci Pollut Res.

[CR74] Pasin D (2018). Non-targeted analysis of new psychoactive substances using mass spectrometric techniques.

[CR75] Pasin D, Cawley A, Bidny S, Fu S (2017). Characterization of hallucinogenic phenethylamines using high-resolution mass spectrometry for non-targeted screening purposes. Drug Test Anal.

[CR76] Pasin D, Cawley A, Bidny S, Fu SL (2017). Current applications of high-resolution mass spectrometry for the analysis of new psychoactive substances: a critical review. Anal Bioanal Chem.

[CR77] Pasin D, Mollerup CB, Rasmussen BS, Linnet K, Dalsgaard PW (2021). Development of a single retention time prediction model integrating multiple liquid chromatography systems: application to new psychoactive substances. Anal Chim Acta.

[CR78] Pettersson Bergstrand M, Beck O, Helander A (2018). Urine analysis of 28 designer benzodiazepines by liquid chromatography–high-resolution mass spectrometry. Clin Mass Spectrom.

[CR79] Płotka-Wasylka J, Szczepańska N, de la Guardia M, Namieśnik J (2015). Miniaturized solid-phase extraction techniques. TrAC, Trends Anal Chem.

[CR80] Pyke JS, Black G, Chen K, Anumol T, Young TM (2019) Simultaneous Targeted Quantitation and suspect screening of environmental contaminants in sewage sludge, Online 5994–0750EN

[CR81] Quinn RA, Nothias L-F, Vining O, Meehan M, Esquenazi E, Dorrestein PC (2017). Molecular networking as a drug discovery, drug metabolism, and precision medicine strategy. Trends Pharmacol Sci.

[CR82] Regueiro J, Negreira N, Berntssen MHG (2016). Ion-Mobility-derived collision cross section as an additional identification point for multiresidue screening of pesticides in fish feed. Anal Chem.

[CR83] Ren S, Hinzman AA, Kang EL, Szczesniak RD, Lu LJ (2015). Computational and statistical analysis of metabolomics data. Metabolomics.

[CR84] Ruttkies C, Schymanski EL, Wolf S, Hollender J, Neumann S (2016). MetFrag relaunched: incorporating strategies beyond in silico fragmentation. J Cheminform.

[CR85] Salgueiro-González N, Castiglioni S, Gracia-Lor E (2019). Flexible high resolution-mass spectrometry approach for screening new psychoactive substances in urban wastewater. Sci Total Environ.

[CR86] Salomone A, Di Corcia D, Negri P (2021). Targeted and untargeted detection of fentanyl analogues and their metabolites in hair by means of UHPLC-QTOF-HRMS. Anal Bioanal Chem.

[CR87] Sana TR, Roark JC, Li X, Waddell K, Fischer SM (2008). Molecular formula and METLIN Personal Metabolite Database matching applied to the identification of compounds generated by LC/TOF-MS. J Biomol Tech.

[CR88] Scalbert A, Brennan L, Fiehn O (2009). Mass-spectrometry-based metabolomics: limitations and recommendations for future progress with particular focus on nutrition research. Metabolomics.

[CR89] Shao Y, Le W (2019). Recent advances and perspectives of metabolomics-based investigations in Parkinson’s disease. Mol Neurodegener.

[CR90] Shevyrin V, Melkozerov V, Nevero A (2015). Identification and analytical characteristics of synthetic cannabinoids with an indazole-3-carboxamide structure bearing a N-1-methoxycarbonylalkyl group. Anal Bioanal Chem.

[CR91] Shevyrin V, Melkozerov V, Eltsov O, Shafran Y, Morzherin Y (2016). Synthetic cannabinoid 3-benzyl-5-[1-(2-pyrrolidin-1-ylethyl)-1H-indol-3-yl]-1,2,4-oxadiazole. The first detection in illicit market of new psychoactive substances. Forensic Sci Int.

[CR92] Skinnider MA, Wang F, Pasin D (2021). A deep generative model enables automated structure elucidation of novel psychoactive substances. Nat Mach Intell.

[CR93] Sleno L (2012). The use of mass defect in modern mass spectrometry. J Mass Spectrom.

[CR94] Smith CA, O'Maille G, Want EJ (2005). METLIN: a metabolite mass spectral database. Ther Drug Monit.

[CR95] Smith CA, Want EJ, O'Maille G, Abagyan R, Siuzdak G (2006). XCMS: processing mass spectrometry data for metabolite profiling using nonlinear peak alignment, matching, and identification. Anal Chem.

[CR96] Solimini R, Minutillo A, Kyriakou C, Pichini S, Pacifici R, Busardo FP (2017). Nails in forensic toxicology: an update. Curr Pharm Des.

[CR97] Stanstrup J, Neumann S, Vrhovšek U (2015). PredRet: prediction of retention time by direct mapping between multiple chromatographic systems. Anal Chem.

[CR98] Stephanson NN, Signell P, Helander A, Beck O (2017). Use of LC–HRMS in full scan-XIC mode for multi-analyte urine drug testing—a step towards a ‘black-box’ solution?. J Mass Spectrom.

[CR99] Steuer AE, Brockbals L, Kraemer T (2019). Metabolomic strategies in biomarker research-new approach for indirect identification of drug consumption and sample manipulation in clinical and forensic toxicology?. Front Chem.

[CR100] Steuer AE, Kaelin D, Boxler MI (2020). Comparative untargeted metabolomics analysis of the psychostimulants 3,4-methylenedioxy-methamphetamine (MDMA), amphetamine, and the novel psychoactive substance mephedrone after controlled drug administration to humans. Metabolites.

[CR101] Streun GL, Elmiger MP, Dobay A, Ebert L, Kraemer T (2020). A machine learning approach for handling big data produced by high resolution mass spectrometry after data independent acquisition of small molecules—proof of concept study using an artificial neural network for sample classification. Drug Test Anal.

[CR102] Suzuki J, El-Haddad S (2017). A review: Fentanyl and non-pharmaceutical fentanyls. Drug Alcohol Depend.

[CR103] Szeremeta M, Pietrowska K, Niemcunowicz-Janica A, Kretowski A, Ciborowski M (2021). Applications of metabolomics in forensic toxicology and forensic medicine. Int J Mol Sci.

[CR104] Talebi M, Schuster G, Shellie RA, Szucs R, Haddad PR (2015). Performance comparison of partial least squares-related variable selection methods for quantitative structure retention relationships modelling of retention times in reversed-phase liquid chromatography. J Chromatogr A.

[CR105] Thermo Fisher Scientific (2021) Compound Discoverer Software. In. https://www.thermofisher.com/au/en/home/industrial/mass-spectrometry/liquid-chromatography-mass-spectrometry-lc-ms/lc-ms-software/multi-omics-data-analysis/compound-discoverer-software.html Accessed 1st August 2021

[CR106] United Nations Office on Drugs and Crime (2020). World Drug Report 2020.

[CR107] United Nations Office on Drugs and Crime (2021) UNODC Early Warning Advisory on New Psychoactive Substances. In. https://www.unodc.org/LSS/Page/NPS Accessed 9th May 2021

[CR108] Urbas A, Schoenberger T, Corbett C, Lippa K, Rudolphi F, Robien W (2018). NPS Data Hub: a web-based community driven analytical data repository for new psychoactive substances. Forensic Chem.

[CR109] Usui K, Murata T, Fujita Y (2018). Direct detection of the psychoactive substance MT-45 in human tissue samples by probe electrospray ionization-tandem mass spectrometry. Drug Test Anal.

[CR110] van der Hooft JJ, Padmanabhan S, Burgess KE, Barrett MP (2016). Urinary antihypertensive drug metabolite screening using molecular networking coupled to high-resolution mass spectrometry fragmentation. Metabolomics.

[CR111] Vandergrift GW, Hessels AJ, Palaty J, Krogh ET, Gill CG (2018). Paper spray mass spectrometry for the direct, semi-quantitative measurement of fentanyl and norfentanyl in complex matrices. Clin Biochem.

[CR112] Vincenti F, Montesano C, Cellucci L (2019). Combination of pressurized liquid extraction with dispersive liquid liquid micro extraction for the determination of sixty drugs of abuse in hair. J Chromatogr A.

[CR113] Vincenti F, Montesano C, Di Ottavio F (2020). Molecular networking: a useful tool for the identification of new psychoactive substances in seizures by LC–HRMS. Front Chem.

[CR114] von Cüpper M, Dalsgaard PW, Linnet K (2020). Identification of new psychoactive substances in seized material using UHPLC–QTOF-MS and an online mass spectral database. J Anal Toxicol.

[CR115] Wang Y, Xiao J, Suzek TO (2011). PubChem's BioAssay Database. Nucleic Acids Res.

[CR116] Wang M, Carver JJ, Phelan VV (2016). Sharing and community curation of mass spectrometry data with Global Natural Products Social Molecular Networking. Nat Biotechnol.

[CR117] Wang Z, Bian L, Mo C (2020). Quantification of aminobutyric acids and their clinical applications as biomarkers for osteoporosis. Commun Bio.

[CR118] Wishart DS, Feunang YD, Marcu A (2017). HMDB 40: the human metabolome database for 2018. Nucleic Acids Res.

[CR119] Woldegebriel M, Derks E (2017). Artificial neural network for probabilistic feature recognition in liquid chromatography coupled to high-resolution mass spectrometry. Anal Chem.

[CR120] Yang JY, Sanchez LM, Rath CM (2013). Molecular networking as a dereplication strategy. J Nat Prod.

[CR121] Yang Y, Li G, Wu D (2020). Recent advances on toxicity and determination methods of mycotoxins in foodstuffs. Trends Food Sci Technol.

[CR122] Yu JS, Seo H, Kim GB, Hong J, Yoo HH (2019). MS-based molecular networking of designer drugs as an approach for the detection of unknown derivatives for forensic and doping applications: a case of NBOMe derivatives. Anal Chem.

[CR123] Zawilska JB (2017). An expanding world of novel psychoactive substances: opioids. Front Psychiatry.

[CR124] Zhang H, Zhang D, Ray K, Zhu M (2009). Mass defect filter technique and its applications to drug metabolite identification by high-resolution mass spectrometry. J Mass Spectrom.

[CR125] Zheng T, Liu L, Aa J (2013). Metabolic phenotype of rats exposed to heroin and potential markers of heroin abuse. Drug Alcohol Depend.

